# The addition of rituximab to CHOP therapy alters the prognostic significance of CD44 expression

**DOI:** 10.1186/1756-8722-7-34

**Published:** 2014-04-16

**Authors:** Xiaolei Wei, Meng Xu, Yongqiang Wei, Fen Huang, Tong Zhao, Xiangzhao Li, Ru Feng, B Hilda Ye

**Affiliations:** 1 Department of Hematology, Nanfang Hospital, Southern Medical University, Guangzhou, China; 2 Department of Pathology, Nanfang Hospital, Southern Medical University, Guangzhou, China; 3 Department of Cell Biology, Albert Einstein College of Medicine, 1300 Morris Park Ave, Bronx, NY 10461, USA

**Keywords:** DLBCL, Prognosis, CHOP, Rituximab, CD44 variant isoforms, Bone marrow involvement

## Abstract

Expression of CD44 splice isoforms has been previously reported to correlate with inferior outcomes in DLBCL patients treated with CHOP therapy. However, it is unclear whether this observation remains valid in the R-CHOP era. In this study, we correlated CD44H and CD44v6 status with survival outcomes among DLBCL patients with an emphasis on the comparison between CHOP- and R-CHOP-treated subgroups. Our results suggest that rituximab has significantly decreased the prognostic value of CD44H. We also observed that the therapeutic benefit of rituximab is largely restricted to CD44H-positive cases in this cohort.

## To the Editor

Although incorporation of rituximab into CHOP (R-CHOP) has dramatically improved the outcome of DLBCL [1-5], approximately 40% of patients still succumb to the disease [6]. One of the prognostic markers studied in the CHOP era is CD44, a transmembrane glycoprotein with many alternative splicing isoforms [7]. Variations in its extracellular domain lead to isoform-specific activities of CD44 in cell adhesion, lymphocyte homing, and cell signaling [7]. In general, CD44 plays a positive role in cell survival and invasiveness, and it is implicated in cancer stem cell maintenance in certain solid tumors [8]. The objective of the current study is to compare the prognostic significance of CD44 isoforms in the CHOP and R-CHOP treatment groups.

This study enrolled 117 de novo DLBCL patients among whom 53 were treated with CHOP and 64 were treated with R-CHOP (Additional file 1; Additional file 2: Table S1). As expected, the incorporation of rituximab markedly improved the overall survival (OS) and event-free survival (EFS) rates (not shown). We used immunohistochemistry (IHC) to examine the expression of CD44H (the standard isoform) and CD44v6 (isoforms containing the variant exon 6) in diagnostic specimens (Additional file 3: Figure S1). Expression of CD44H and CD44v6 was detected in 65.0% and 34.2% of patients, respectively, with strong correlation to each other (Spearman’s correlation, r = 0.423, *p* < 0.001). The baseline clinical features were not significantly different between the CD44H+ and CD44H- patients. The CD44v6+ and CD44v6- cases were also very comparable (Additional file 2: Table S2).

**Figure 1:**
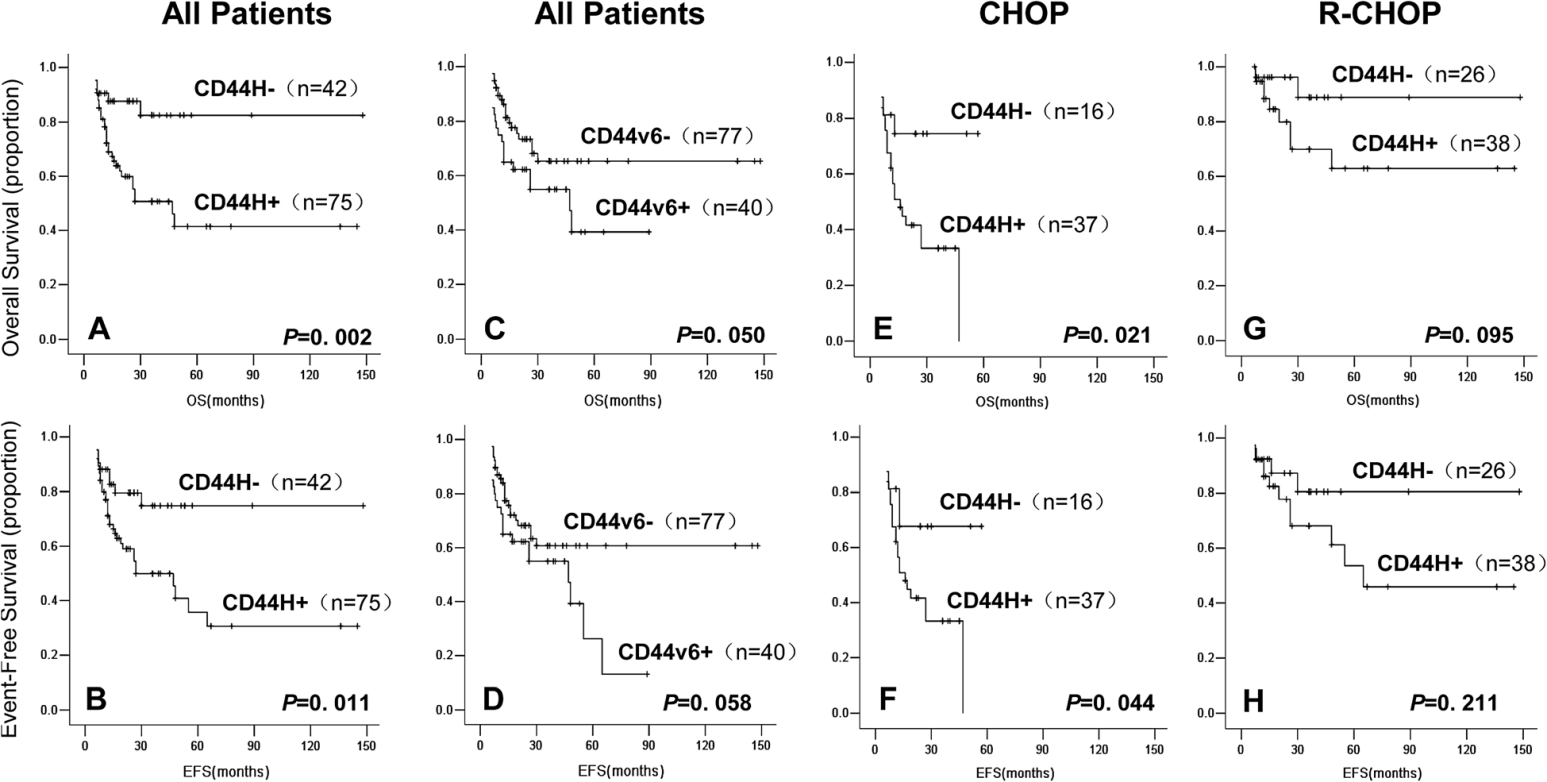
**Overall survival (OS) and event-free survival (EFS) according to CD44H and CD44v6 expression status and type of therapy.** The Kaplan-Meier method was used to estimate the OS and EFS distributions with the log-rank test performed to compare the survival curves. OS **(A and C)** and EFS **(B and D)** of all patients were analyzed based on their CD44H **(A and B)** and CD44v6 **(C and D)** status. OS **(E and G)** and EFS **(F and H)** according to CD44H expression status were also analyzed in the CHOP **(E and F)** and R-CHOP **(G and H)** treatment groups separately.

**Figure 2:**
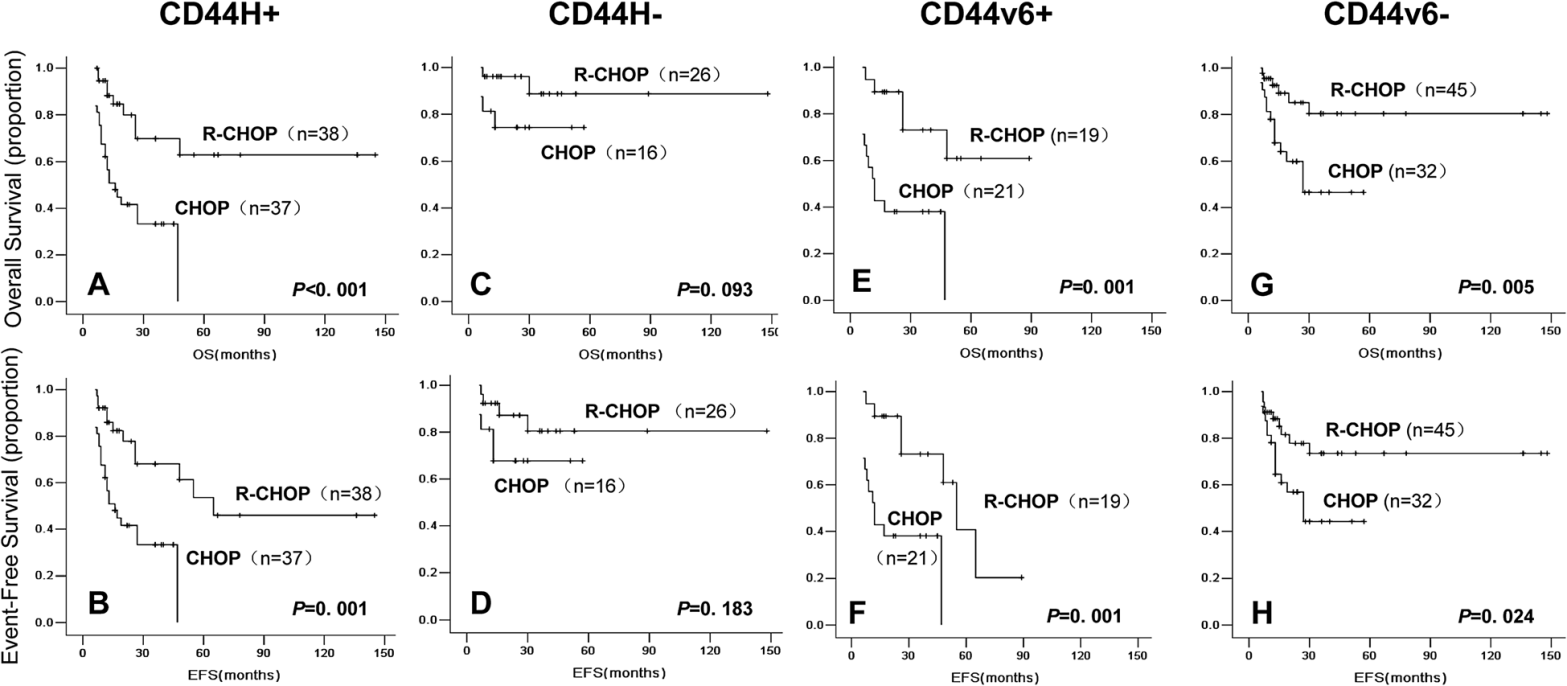
**Rituximab-associated survival benefits based on CD44H and CD44v6 expression status.** The Kaplan-Meier method was used to estimate the OS and EFS distributions with the log-rank test performed to compare the survival curves. For cases that were either CD44H+ **(A and B)** or CD44H- **(C and D)**, survival outcomes after CHOP and R-CHOP treatments were compared for OS **(A and C)** and EFS **(B and D)**. CHOP versus R-CHOP comparison was also made for OS **(E and G)** and EFS **(F and H)** among the CD44V6+ **(E and F)** and CD44v6- **(G and H)** subgroups.

In the entire cohort of 117 patients, CD44H positivity strongly correlated with poor OS (*p* = 0.002, Figure 1A) and EFS (*p* = 0.011, Figure 1B) outcomes. Specifically, the 5-year OS rates in the CD44H+ and CD44H- subgroups were 82% and 41%, respectively. CD44v6 positivity also correlated with poor prognosis, although the trend was only marginally significant (OS: *p* = 0.050; EFS: *P* = 0.058, Figure 1C and D). Nevertheless, because CD44v6 showed an IPI-independent survival impact in multi-variable analysis (Additional file 2: Table S3), the relatively weak survival association based on the Kaplan-Meier estimates likely reflects the low frequency of CD44v6 expression and hence a greater sample size requirement. CD44v6 did not show any prognostic value when the cohort was divided into treatment subgroups (not shown). The negative prognostic value for CD44H detected among all patients could also be observed in the CHOP subgroup (OS: *p* = 0.021; EFS: *P* = 0.044, Figure 1E and F), but not the R-CHOP subgroup (OS: *p* = 0.095; EFS: *P* = 0.211, Figure 1G and H). Because the OS response was very similar among all R-CHOP-treated cases and CHOP-treated CD44H- patients, we reasoned that the extremely unfavorable response to CHOP among CD44H-positive patients may have been specifically ameliorated by rituximab. To test this notion, the rituximab-associated survival benefit was examined in patient subgroups of different CD44 expression status. For CD44H, although rituximab substantially improved the outcome for CD44H+ patients (OS: *p* < 0.001; EFS: *P* = 0.001, Figure 2A and B), the impact of this agent was insignificant for the CD44H- cases (OS: *p* = 0.093; EFS: *P* = 0.183, Figure 2C and D). Interestingly, this phenomenon appeared to be specific to CD44H because the rituximab-associated survival benefit was significant irrespective of the CD44v6 status (Figure 2E to H).

Possibly due to the use of different antibodies and different IHC staining/scoring methods, there have been some controversial observations on the prognostic importance of CD44 in CHOP-treated DLBCL patients. In agreement with the majority of published studies [9-11], we have observed a negative survival impact of CD44H and CD44v6 expression in our entire study cohort (Figure 1 and Additional file 2: Table S3) as well as the CHOP treatment group (Figure 1E and F), although there were differences between these two markers. As the first study aimed to examine CD44 isoform expression in the R-CHOP era, our data suggest that rituximab has decreased the prognostic significance of CD44H, while the impact of rituximab on CD44v6 awaits future studies of larger cohorts. We also observed that the rituximab-associated survival benefit was profound among CD44H-positive cases but fairly limited among the CD44H-negative subgroup.

## Competing interests

The authors declare no conflicts of interest.

## Authors’ contributions

FR and BHY designed the study and analyzed and interpreted the data. WXL and XM collected and analyzed data. WYQ and HF collected data. ZT and LXZ provided study material and helped with the IHC staining. BHY, WXL and FR wrote the manuscript. All authors read and approved the final manuscript.

## Supplementary Material

Additional file 1 Information on Patients and Methods. Click here for file

Additional file 2: Table S1 Clinical features and CD44 variant expression in the CHOP and RCHOP groups. **Table S2.** Patient characteristics according to CD44H and CD44v6 expression status. **Table S3.** Prognostic factors and multivariable survival analysis. Click here for file

Additional file 3: Figure S1 Representative immunohistochemical staining of DLBCL samples for CD44H and CD44v6 expression. (A, C), negative control stain using isotype-matched Abs. (B) CD44H staining in apositive case. (D) CD44v6 staining in a positive case. Click here for file

## References

[B1] Coiffier B Lepage E Briere J Herbrecht R Tilly H Bouabdallah R Morel P Van Den Neste E Salles G Gaulard P Reyes F Lederlin P Gisselbrecht C CHOP chemotherapy plus rituximab compared with CHOP alone in elderly patients with diffuse large-B-cell lymphoma N Engl J Med 2002 346 4 235 242 10.1056/NEJMoa011795 11807147

[B2] Habermann TM Weller EA Morrison VA Gascoyne RD Cassileth PA Cohn JB Dakhil SR Woda B Fisher RI Peterson BA Horning SJ Rituximab-CHOP versus CHOP alone or with maintenance rituximab in older patients with diffuse large B-cell lymphoma J Clin Oncol: Official J Am Soc Clin Oncol 2006 24 19 3121 3127 10.1200/JCO.2005.05.1003 16754935

[B3] Pfreundschuh M Schubert J Ziepert M Schmits R Mohren M Lengfelder E Reiser M Nickenig C Clemens M Peter N Bokemeyer C Eimermacher H Ho A Hoffmann M Mertelsmann R Trümper L Balleisen L Liersch R Metzner B Hartmann F Glass B Poeschel V Schmitz N Ruebe C Feller AC Loeffler M German High-Grade Non-Hodgkin Lymphoma Study Group (DSHNHL) Six versus eight cycles of bi-weekly CHOP-14 with or without rituximab in elderly patients with aggressive CD20+ B-cell lymphomas: a randomised controlled trial (RICOVER-60) Lancet Oncol 2008 9 2 105 116 10.1016/S1470-2045(08)70002-0 18226581

[B4] Pfreundschuh M Trumper L Osterborg A Pettengell R Trneny M Imrie K Ma D Gill D Walewski J Zinzani PL Stahel R Kvaloy S Shpilberg O Jaeger U Hansen M Lehtinen T López-Guillermo A Corrado C Scheliga A Milpied N Mendila M Rashford M Kuhnt E Loeffler M MabThera International Trial Group CHOP-like chemotherapy plus rituximab versus CHOP-like chemotherapy alone in young patients with good-prognosis diffuse large-B-cell lymphoma: a randomised controlled trial by the MabThera International Trial (MInT) Group Lancet Oncol 2006 7 5 379 391 10.1016/S1470-2045(06)70664-7 16648042

[B5] Cang S Mukhi N Wang K Liu D Novel CD20 monoclonal antibodies for lymphoma therapy J Hematol Oncol 2012 5 64 10.1186/1756-8722-5-64 23057966 PMC3479003

[B6] Sehn LH Paramount prognostic factors that guide therapeutic strategies in diffuse large B-cell lymphoma Hematology Am Soc Hematol Educ Program 2012 2012 402 409 23233611 10.1182/asheducation-2012.1.402

[B7] Ponta H Sherman L Herrlich PA CD44: from adhesion molecules to signalling regulators Nat Rev Mol Cell Biol 2003 4 1 33 45 10.1038/nrm1004 12511867

[B8] Guo W Frenette PS Alternative CD44 splicing in intestinal stem cells and tumorigenesis Oncogene 2013 10.1038/onc.2013.260 23831568

[B9] Horst E Meijer CJ Radaszkiewicz T Ossekoppele GJ Van Krieken JH Pals ST Adhesion molecules in the prognosis of diffuse large-cell lymphoma: expression of a lymphocyte homing receptor (CD44), LFA-1 (CD11a/18), and ICAM-1 (CD54) Leukemia 1990 4 8 595 599 1974938

[B10] Inagaki H Banno S Wakita A Ueda R Eimoto T Prognostic significance of CD44v6 in diffuse large B-cell lymphoma Mod Pathol 1999 12 5 546 552 10349995

[B11] Drillenburg P Wielenga VJ Kramer MH van Krieken JH Kluin-Nelemans HC Hermans J Heisterkamp S Noordijk EM Kluin PM Pals ST CD44 expression predicts disease outcome in localized large B cell lymphoma Leukemia 1999 13 9 1448 1455 10.1038/sj.leu.2401490 10482998

