# Supplementary material for: The addition of rituximab to CHOP therapy alters the prognostic significance of CD44 expression
Source: J Hematol Oncol. 2014 Apr 16;7:34. doi: 10.1186/1756-8722-7-34 (PMC4022142; doi:10.1186/1756-8722-7-34)
Supplement: Additional file 1 — Information on Patients and Methods. [file 1756-8722-7-34-S1.doc]

**Additional file 1, Wei et al**

**Information on Patients and Methods**

**Patient Population**

Included in this retrospective study are 120 patients diagnosed with de novo DLBCL between 1998 and 2008 in Nan Fang Hospital, Guangzhou, China. Patients were included in the study if they were ≥ 18 years of age with a biopsy-proven diagnosis of DLBCL according to the current WHO criteria , had unilateral BM aspirate and biopsy at the time of diagnosis and had complete clinical data. Patients were excluded if they were HIV-positive, or had various other types of lymphoma, including primary mediastinal, central nervous system, intravascular and testicular lymphomas. All patients were staged based on the Ann Arbor staging system and assigned IPI scores using the original definition . Among the 120 patients, 53 were treated with CHOP, 64 were treated with R-CHOP, either alone or in combination with surgery and/or involved-field radiation. Three patients were treated with other types of therapies and were included in the CD44 expression analysis but excluded from prognostic studies. The study was approved by the institutional review board of the Nan Fang Hospital.

**Immunohistochemistry (IHC)**

Sections from formalin-fixed and paraffin-embedded diagnostic samples were collected for histological review and immunohistochemical analysis. IHC was carried out using a peroxidase-conjugated labeled dextran polymer method as previously described . Rabbit polyclonal antibody for BCL6 (N3) was from Santa Cruz Biotechnology. The mouse monoclonal antibody (mAb) for CD10 (Clone 56C6) was from Novocastra, mAb for MUM1 (clone MUM1p) was from Dako, and mAbs for CD44H (clone 156-3C11) and CD44v6 (clone VVF-7) were from ZSGB-BIO, Beijing, China. CD44 isoform expression was scored using a semiquantitative method as described by Fromowitz et al Briefly, 500 cells in 5 well-preserved areas were scored for overall staining intensity and the percentage of the positively stained cells. Intensity was graded as negative (0) (no evidence of staining), low (1) (weak positive staining), moderate (2) (positive staining), and intense (3) (strong positive staining). Proportional staining was classified as negative (0): < 5%, (1): 5-25%, (2): 26-50%, (3): 51-75%, (4) : >75%. A composite score ranging from 0 to 7 was then calculated as the sum of intensity and percentage scores. The cut-off for CD44H positivity was >= 1 while the cut-off for CD44v6 was >= 2. For COO (GCB/non-GCB) sub-classification, CD10, BCL6, and Mum1 positivity was defined according to Hans et al .

**Statistical analysis**

Statistical analyses were performed using the SPSS software, version 13.0. The Spearman test was used to analyze relationships between the CD44H and CD44v6 expression. The Mann-Whitney U-test was applied to assess the difference between group means. Event free survival (EFS) was calculated from the date of diagnosis to the date of documented disease progression, relapse or death from any cause or to the date on which the study was stopped. Overall survival (OS) was calculated from the date of diagnosis until death from any cause or the last follow-up. The Kaplan-Meier method was used to estimate the OS and EFS distributions with the log-rank test performed to compare the survival curves. Univariable and multivariable modeling of OS and EFS was based on the Cox regression analysis. *P* < 0.05 was considered to indicate statistical significance.

# References

1. Swerdlow SH, Campo E, Harris NL, Jaffe ES, Pileri SA, Stein H, Thiele J, Vardiman JW, IARC: **WHO Classification of Tumours of Haematopoietic and Lymphoid Tissues.** In. Geneva, Switzerland: World Health Organization; 2008

2. Lister TA, Crowther D, Sutcliffe SB, Glatstein E, Canellos GP, Young RC, Rosenberg SA, Coltman CA, Tubiana M: **Report of a committee convened to discuss the evaluation and staging of patients with Hodgkin's disease: Cotswolds meeting.** *Journal of clinical oncology : official journal of the American Society of Clinical Oncology* 1989, **7:**1630-1636.

3. Project. TIN-HsLPF: **A predictive model for aggressive non-Hodgkin's lymphoma. The International Non-Hodgkin's Lymphoma Prognostic Factors Project.** *The New England journal of medicine* 1993, **329:**987-994.

4. Li HL, Huang XP, Zhou XH, Ji TH, Wu ZQ, Wang ZQ, Jiang HY, Liu FR, Zhao T: **Correlation of seven biological factors (Hsp90a, p53, MDM2, Bcl-2, Bax, Cytochrome C, and Cleaved caspase3) with clinical outcomes of ALK+ anaplastic large-cell lymphoma.** *Biomedical and environmental sciences : BES* 2011, **24:**630-641.

5. Fromowitz FB, Viola MV, Chao S, Oravez S, Mishriki Y, Finkel G, Grimson R, Lundy J: **ras p21 expression in the progression of breast cancer.** *Human pathology* 1987, **18:**1268-1275.

6. Hans CP, Weisenburger DD, Greiner TC, Gascoyne RD, Delabie J, Ott G, Muller-Hermelink HK, Campo E, Braziel RM, Jaffe ES, et al: **Confirmation of the molecular classification of diffuse large B-cell lymphoma by immunohistochemistry using a tissue microarray.** *Blood* 2004, **103:**275-282.
