# Supplementary material for: The addition of rituximab to CHOP therapy alters the prognostic significance of CD44 expression
Source: J Hematol Oncol. 2014 Apr 16;7:34. doi: 10.1186/1756-8722-7-34 (PMC4022142; doi:10.1186/1756-8722-7-34)
Supplement: Additional file 2: Table S1 — Clinical features and CD44 variant expression in the CHOP and RCHOP groups. Table S2. Patient characteristics according to CD44H and CD44v6 expression status. Table S3. Prognostic factors and multivariable survival analysis. [file 1756-8722-7-34-S2.pdf]

Wei et al.

Additional file 2.

Table S1. Clinical features and CD44 variant expression in the CHOP and R-CHOP groups.

Characteristics	*N*	CHOP		R-CHOP		*P*
		n	%	n	%	
Gender						0.380
Female	50	25	47.2	25	39.1	
Male	67	28	52.8	39	60.1	
Age(years)						0.487
<60y	71	34	64.2	37	57.8	
≥60y	46	19	35.8	27	42.2	
Systemic symptoms						0.360
A	78	33	62.3	45	70.3	
B	39	20	37.3	19	29.7	
BM involvement						0.004
Yes	22	16	30.2	6	9.4	
No	95	37	69.8	58	90.6	
LDH levels						0.015
Normal	71	26	47.2	45	70.3	
High	46	27	52.8	19	29.7	
Ann Arbor stage						0.884
I or II	45	20	37.3	25	39.1	
III or IV	72	33	62.3	39	60.9	
No. of extranodal sites						0.498
0-1	81	35	66.0	46	71.9	
≥2	36	18	34.0	18	28.1	
IPI score						0.104
0-1	54	20	37.7	34	53.1	
2	26	13	24.5	13	20.3	
3	18	7	13.2	11	17.2	
4-5	19	13	24.5	6	9.4	
COO subtype						0.707
GCB	42	20	37.7	22	34.4	
NON-GCB	75	33	62.3	42	65.6	
CD44H						0.243
-	42	16	30.2	26	40.6	
+	75	37	69.8	38	59.4	
CD44v6						0.261
-	77	32	60.4	45	70.3	
+	40	21	39.6	19	29.7	

Mann-Whitney U test was applied to assess the difference in distribution between the CHOP and R-CHOP groups.

Additional file 2.

Table S2.Patient characteristics according to CD44H and CD44v6 expression status.

Characteristics	*n*	CD44H		*P*	CD44v6		*P*
		—	+		—	+	
Gender				0.173			0.113
Female	53(44.2%)	15	38		39	14	
Male	67(55.8%)	27	40		40	27	
Age(years)				0.276			0.309
<60y	72(60.0%)	28	44		50	22	
≥60y	48(40.0%)	14	34		29	19	
Systemic symptoms				0.887			0.782
A	81(67.5%)	28	53		54	27	
B	39(32.5%)	14	25		25	14	
IPI score				0.130			0.642
0-2	83(72.5%)	33	50		55	28	
3-5	37(27.5%)	9	28		24	13	
Bone marrow involvement				0.014			0.365
Yes	23(19.2%)	3	20		17	6	
No	97(80.8%)	39	58		62	35	
Lactate dehydrogenase				0.218			0.193
Normal	74(61.7%)	29	45		52	22	
High	46(38.3%)	13	33		27	19	
Ann Arbor stage				0.756			0.309
or	48(40.0%)	16	32		29	19	
or	72(60.0%)	26	46		50	22	
No. of extranodal sites				0.421			0.273
0-1	83(69.2%)	31	52		52	31	
≥2	37(30.8%)	11	26		27	10	
Cell-of-origin subtype				0.116			0.499
GCB	43(35.8%)	19	24		30	13	
NON-GCB	77(64.2%)	23	54		49	28	
Treatment				0.243			0.261
CHOP	53(44.2%)	16	37		32	21	
R-CHOP	64(53.3%)	26	38		45	19	

Note: Three patients that were treated with therapies other than CHOP and R-CHOP were included in this table but excluded from the prognostic studies.

Additional file 2.

Table S3.Prognostic factors and multivariable survival analysis.

Prognostic factors	Multivariable analysis		
	RR	95%CI	*P*
**Analysis of the entire cohort(n=117)**			
**Overall survival**			
IPI (High:3~5 vs Low:0~2)	2.9	2.0-4.1	**<0.001**
CD44H (PosvsNeg)	1.3	0.48-3.4	0.618
CD44v6 (PosvsNeg)	2.4	1.2-4.7	**0.017**
**Event-free survival**			
IPI (High:3~5 vs Low:0~2)	2.4	1.8-3.3	**<0.001**
CD44H (Pos vs Neg)	1.1	0.47-2.5	0.861
CD44v6 (Pos vs Neg)	2.2	1.1-4.1	**0.019**
**Analysis of the CHOP group (n=53)**			
**Overall survival**			
IPI (High:3~5 vs Low:0~2)	2.6	1.7-4.0	**<0.001**
CD44H (PosvsNeg)	0.93	0.25-3.4	0.909
CD44v6 (PosvsNeg)	2.1	0.9-5.3	0.098
**Event-free survival**			
IPI (High:3~5 vs Low:0~2)	2.5	1.7-3.8	**<0.001**
CD44H (PosvsNeg)	0.76	0.22-2.6	0.664
CD44v6 (PosvsNeg)	2.1	0.87-5.2	0.096
**Analysis of the R-CHOP group (n=64)**			
**Overall survival**			
IPI (High:3~5 vs Low:0~2)	2.9	1.5-5.5	**0.002**
CD44H (PosvsNeg)	1.6	0.3-8.7	0.579
CD44v6 (PosvsNeg)	2.3	0.6-8.5	0.226
**Event-free survival**			
IPI (High:3~5 vs Low:0~2)	2.0	1.5-4.5	**0.003**
CD44H (PosvsNeg)	1.3	0.38-4.4	0.691
CD44v6 (PosvsNeg)	2.0	0.69-5.9	0.201
