# Supplementary material for: The addition of rituximab to CHOP therapy alters the prognostic significance of CD44 expression
Source: J Hematol Oncol. 2014 Apr 16;7:34. doi: 10.1186/1756-8722-7-34 (PMC4022142; doi:10.1186/1756-8722-7-34)
Supplement: Additional file 3: Figure S1 — Representative immunohistochemical staining of DLBCL samples for CD44H and CD44v6 expression. (A, C), negative control stain using isotype-matched Abs. (B) CD44H staining in apositive case. (D) CD44v6 staining in a positive case. [file 1756-8722-7-34-S3.pdf]

Additional File 3, Wei et al.

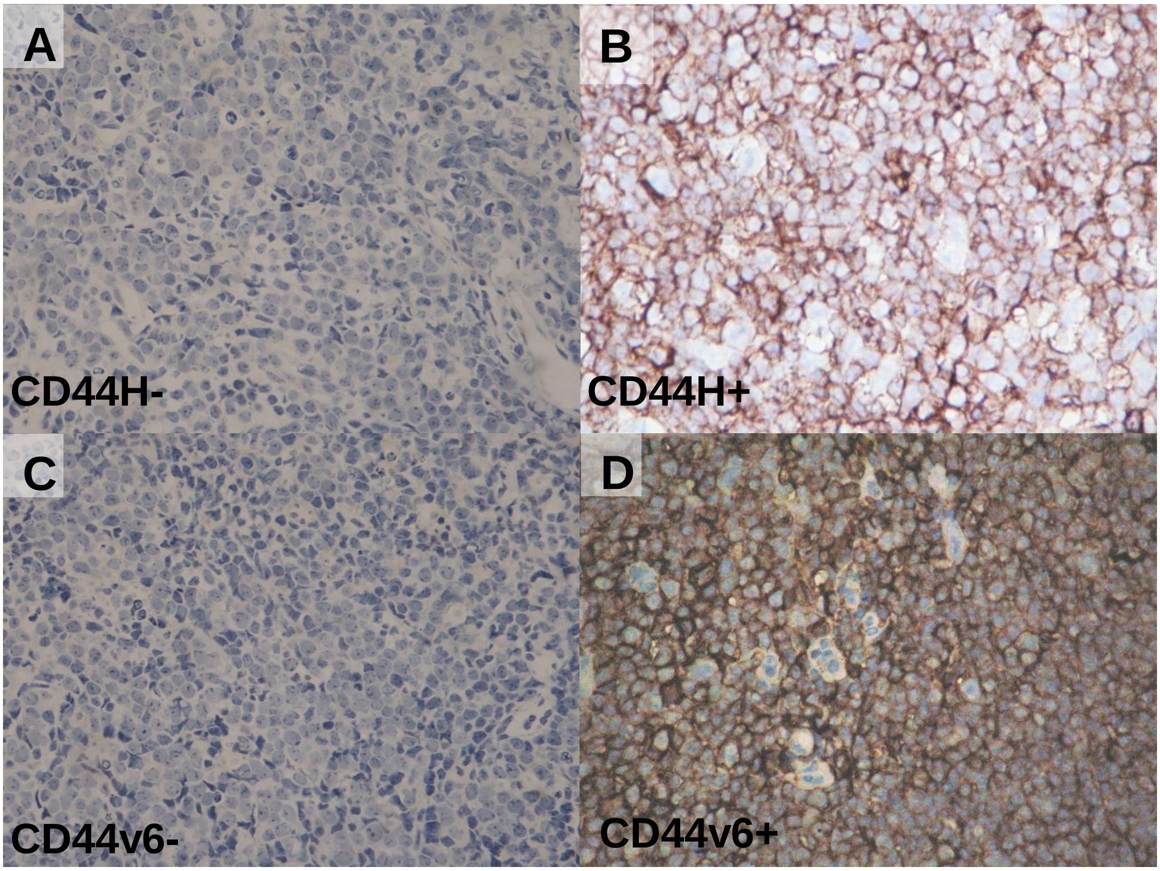

Figure A1. Representative immunohistochemical staining of DLBCL samples for CD44H and CD44v6 expression. (**A, C**), negative control stain using isotype-matched Abs. (**B**) CD44H staining in a positive case. (**D**) CD44v6 staining in a positive case.
